# Impact of HLA Alleles on COVID-19 Severity in Kidney Transplant Recipients: A Single-Center Study

**DOI:** 10.7759/cureus.67881

**Published:** 2024-08-26

**Authors:** Necip Altundaş, Eda Balkan, Murat Kızılkaya, Nurhak Aksungur, Salih Kara, Ercan Korkut, Can Sevinç, Gürkan Öztürk

**Affiliations:** 1 Department of General Surgery, Atatürk University, Erzurum, TUR; 2 Department of Medical Biology, Atatürk University, Erzurum, TUR; 3 Department of Internal Medicine, Atatürk University, Erzurum, TUR

**Keywords:** transplant, kidney, covid-19, alleles, hla

## Abstract

Introduction

The coronavirus disease 2019 (COVID-19) pandemic has significantly impacted global health, particularly affecting vulnerable populations, such as organ transplant recipients. Human leukocyte antigens (HLAs) play a critical role in immune response regulation, and understanding their association with COVID-19 can provide insights into disease susceptibility and severity. This study aims to explore the association between HLA allele variability and COVID-19 susceptibility and severity among kidney transplant recipients.

Methods

In 2023, we conducted a study on 73 kidney transplant recipients who tested positive for COVID-19 via polymerase chain reaction. This study included assessments of clinical status, immunosuppressive drug levels, HLA allele frequencies, and donor-recipient tissue compatibility. Molecular analyses were performed using sequence-specific oligonucleotide typing, and statistical analysis was conducted using IBM SPSS Statistics, version 20.0 (IBM Corp., Armonk, NY).

Results

Among the participants, 31 were hospitalized and 42 were treated as outpatients. Significant differences were observed in HLA allele distributions, particularly the HLA-A*11 allele, which was more prevalent in outpatient-treated patients, suggesting a potential protective effect. No significant age differences were observed between hospitalized and outpatient groups. Serum tacrolimus levels were notably higher in outpatients. Statistical analyses revealed significant associations between certain HLA groups and the severity of COVID-19 infection.

Conclusions

This study highlights the importance of HLA allele compatibility in influencing the clinical outcomes of COVID-19 in kidney transplant recipients. The findings suggest that specific HLA alleles may reduce susceptibility or moderate the severity of COVID-19, indicating a need for broader genetic studies across diverse populations to validate these observations and improve management strategies for transplant recipients during pandemics.

## Introduction

Severe acute respiratory syndrome coronavirus-2 (SARS-CoV-2) was identified in December 2019 as the causative agent of coronavirus disease 2019 (COVID-19) [1]. Since its emergence, the COVID-19 pandemic has profoundly impacted the global economy and healthcare systems. Identifying genetic, epigenetic, and environmental factors that contribute to susceptibility to COVID-19 is crucial [2]. Human leukocyte antigens (HLAs), representing one of the most polymorphic systems within the human genome, consist of 33,490 alleles across three gene classes (classes I, II, and III). HLA-encoded molecules play key roles in antigen presentation, inflammation, and both innate and adaptive immune responses [3,4].

HLAs are critical for understanding viral mechanisms and elucidating COVID-19 pathogenesis. Research into the antigen presentation of HLA molecules is essential for clarifying the pathogenesis of COVID-19, about which current knowledge remains limited. The association between HLA-B46:01 and SARS was first noted in 2003 [5-7]. Subsequent studies in diverse populations have identified common alleles (HLA-DRB115 and HLA-A30:02) and have suggested that alleles, such as HLA-DRB104 and HLA-DRB1*08, may increase disease severity. Although these alleles have been linked to COVID-19 and increased mortality, the case numbers have been small, underscoring the need for more extensive studies [8-10].

The COVID-19 pandemic significantly disrupted solid organ transplantation, resulting in decreased transplantations. Transplant recipients are considered more susceptible to COVID-19 due to the immunosuppressive drugs they must take. While understanding the immunological response to ribonucleic acid viruses is critical in kidney transplantation, researching genetic factors such as HLAs in transplant patients affected by COVID-19 is also paramount.

Throughout the pandemic, extensive efforts worldwide have been directed toward discovering effective COVID-19 treatments. Various therapeutic immunomodulators have been explored and used; however, most protocols have excluded recipients of solid organ transplants [11]. Individual variations in HLA alleles can influence viral infections, including COVID-19. Previous research has assessed the potential relationships between HLA alleles and the severity and susceptibility to the disease [6,7].

Our study aims to explore the genetic, clinical, and demographic factors influencing kidney transplant recipients to determine the impact of HLA alleles and donor-recipient tissue compatibility on susceptibility to and severity of COVID-19 infection. Additionally, this study seeks to examine the immunological effects of the levels of immunosuppressive drugs used by patients with COVID-19 infection.

## Materials and methods

In 2023, our study included 73 kidney transplant recipients who tested positive for COVID-19 using the polymerase chain reaction (PCR) test. These patients presented to the center with high fever, body aches, fatigue, and cough symptoms. We excluded all other infections at the time of diagnosis and included only those without active rejection attacks and with reliable data availability. The diagnosis and treatment procedures for COVID-19 were conducted at the Training and Research Hospital. Of the 73 patients, 31 were hospitalized for treatment and 42 were managed as outpatients. All participants were adult kidney transplant recipients, averaging 42.3 years of age.

Follow-up and data access for all included patients were unproblematic, allowing complete data analysis. The organ transplantation accreditation procedures were conducted in the Tissue Typing Laboratory of our study center, which is accredited by the Turkish Ministry of Health. The present study was approved by the Atatürk University Faculty of Medicine Ethics Committee (approval number B.30.2.ATA.0.01.00/3/25). Our study is retrospective.

Patient follow-up and observation

The clinical status of COVID-19 infections was assessed using the five-stage clinical progression scale defined by the World Health Organization (WHO) COVID-19 study group in August 2020. These stages include non-infected, outpatient with mild disease, hospitalized with moderate disease, hospitalized with severe disease, and hospitalized with exitus. Patients scoring 4 or 5 on this scale required hospitalization. A score of 4 indicated the need for supportive care, a score of 5 was assigned to patients requiring oxygen, and scores of 6 or more indicated the need for intubation and intensive care.

During their hospital stay, 30 COVID-19-positive kidney transplant recipients had their anti-proliferative medications (mycophenolate mofetil, CellCept) discontinued. Their immunosuppression regimen was adjusted to tacrolimus at 4/6 mg and steroids at 5 mg. Meanwhile, the 42 outpatients continued their regular doses of anti-proliferative medications, tacrolimus, and steroids as previously prescribed.

We recorded all participants’ etiological, demographic, and clinical data (including routine biochemistry), medication usage, and COVID-19 Radiological Assessment Scoring System (CO-RADS), with detailed recordings for hospitalized patients.

Molecular analyses

DNA Isolation

We collected 5 cc of blood from each patient in ethylenediaminetetraacetic acid tubes. DNA isolation was performed using the QIAGEN EZ1 DNA Blood 200 µL Kit (Hilden, Germany) according to the manufacturer’s protocol. We pipetted 200 µL of blood into 2 mL sample tubes, ensuring thorough suspension before pipetting. EZ1 DNA Blood Cartridges were then placed in the cartridge slots of the EZ1 device, and 1.5 mL DNA elution tubes were prepared and positioned appropriately. After setting up the EZ1 device with the correct protocol, the process was started, which took approximately 18 minutes. The elution tubes containing the purified DNA were stored at −20°C until required for HLA testing.

Class I and II Typing

HLA typing utilized the DNA-based sequence-specific oligonucleotide (SSO) method with The LIFECODES HLA Typing Kit (Immucor, Inc., Norcross, GA, USA). We prepared samples at low resolution for both HLA classes I and II using the SSO method focused on HLA-DR alleles and analyzed them on the Luminex Assay (Luminex Corp., Austin, TX, USA).

SSO Method

For the SSO method, 4 µL of patient DNA was transferred to PCR tubes. We added 3 µL of Master Mix and 0.2 µL of Taq polymerase from The LIFECODES HLA Typing Kit to each sample, along with 6 µL of sterile distilled water. After the initial PCR, products were transferred to 5 µL Eppendorf tubes. Magnetic beads from the kit were then sonicated for one to two minutes at 56°C. We added 16 µL of sonicated magnetic beads to the PCR products and conducted a second PCR. Streptavidin was prepared in the dark (0.85 µL of streptavidin added to 170 µL buffer per sample) and added to the PCR products, which were then loaded into the Luminex device according to the HLA protocol. Results were analyzed using the MatchIT DNA program (Immucor, Inc.), comparing each sample’s probe values against the kit’s threshold median fluorescence intensity.

Statistical analyses

Data collected in the study were analyzed using IBM SPSS Statistics, version 20.0 (IBM Corp., Armonk, NY). We presented descriptive statistics as means ± standard deviation, medians, and range (minimum to maximum) for continuous variables and as counts (n) and percentages (%) for categorical variables. The normality of continuous variables was tested using the Shapiro-Wilk W-test, the Kolmogorov-Smirnov test, Q-Q plots, and measures of skewness and kurtosis.

In our study, we used the independent samples t-test for variables with a normal distribution to compare two independent groups and the Mann-Whitney U test for variables that did not meet parametric assumptions. To compare the two proportions, we applied the chi-square test. For 2 × 2 comparisons, we used the Pearson chi-square test when the expected count was greater than 5, Yates’ correction for continuity when the expected count was between 3 and 5, and Fisher’s exact test when the expected count was less than 3. For categorical variables in matrices larger than 2 × 2, we used the Pearson chi-square test when the expected count was greater than 5 and the Fisher-Freeman-Halton test when the expected count was less than 5. A p-value of <0.05 was considered statistically significant. Additionally, the Bonferroni correction was applied to reduce the likelihood of identifying spurious associations.

## Results

**Figure 1:**
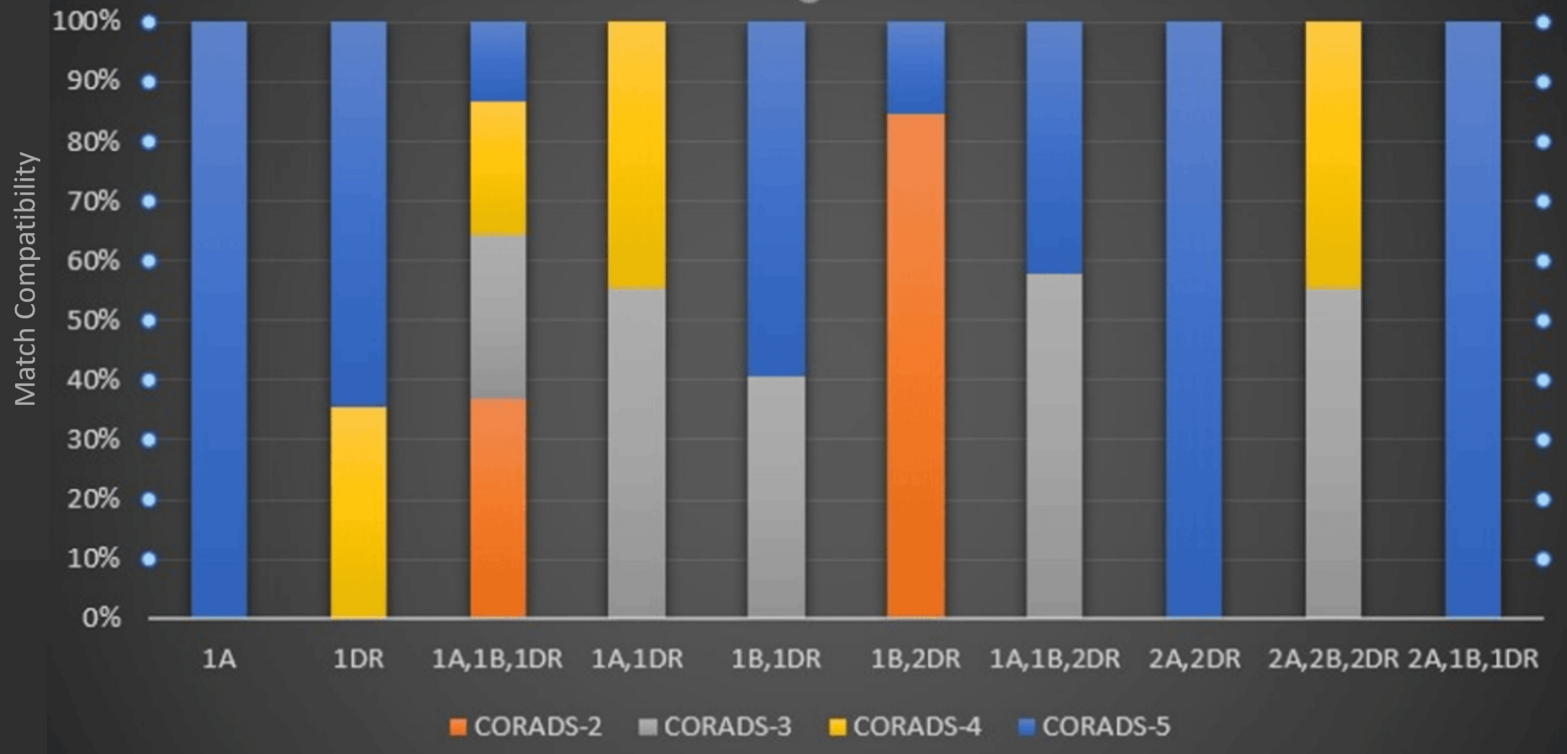
Relationship between the recipient-donor match compatibility and CO-RADS CO-RADS, COVID-19 Radiological Assessment Scoring System

Among the 73 kidney transplant recipients, 31 were hospitalized and 42 received outpatient treatment. We assessed age, gender, etiology, post-transplant medications, routine biochemistry tests, serum tacrolimus levels, HLA allele frequencies, HLA match rates between donor and recipient, and CO-RADS evaluations in hospitalized patients with COVID-19. No significant differences were observed between hospitalized and outpatient COVID-19-positive transplant recipients in terms of routine biochemistry results and medications used (p > 0.05; Tables 1-4). However, serum tacrolimus levels were significantly higher in outpatient-treated patients, 1.4 times higher than in hospitalized patients (p < 0.05; Figure 1).

**Table 1:** Demographic and clinical characteristics of kidney transplant recipients by COVID-19 hospitalization status COVID-19, coronavirus disease 2019; EPO, erythropoietin; NA, not applicable

Demographic and clinical data	Hospitalized for COVID-19		Control
	Yes	No	
Mean age in years			
Male	40.4	39.5	46.7
Female	37.6	34.3	44
Gender			
Male, n (%)	24 (77.42%)	30 (71.43%)	28 (56%)
Female, n (%)	7 (22.58%)	12 (28.57%)	22 (44%)
Etiology			
Hypertension, n (%)	15 (74.19%)	27 (71.84%)	NA
Diabetes, n (%)	8 (28.81%)	9 (18.51%)	NA
Membranoproliferative, n (%)	1 (3.23%)	1 (2.05%)	NA
Idiopathic, n (%)	1 (3.23%)	1 (2.05%)	NA
History of pulmonary tuberculosis, n (%)	1 (3.23%)	1 (2.05%)	NA
Glomerulonephritis, n (%)	2 (6.45%)	1(2.05%)	NA
Right nephrectomy at age 6, n (%)	1 (3.23%)	1 (2.05%)	NA
Single kidney, n (%)	1 (3.23%)	-	NA
Polycystic kidney, n (%)	1 (3.23%)	2 (6.72%)	NA
Drugs used			
Cyclosporine, n (%)	1 (3.23%)	2 (4.55%)	NA
Mycophenolic, n (%)	29 (93.55%)	35 (94.55%)	NA
Valganciclovir, n (%)	30 (96.77%)	35 (94.55%)	NA
Amlodipine, n (%)	17 (54.84%)	20 (60.08%)	NA
Doxazosin, n (%)	5 (16.13%)	3 (8.45%)	NA
Insulin glargine, n (%)	4 (12.9%)	4 (10.96%)	NA
Tacrolimus, n (%)	29 (93.55%)	37 (96.32%)	NA
Prednisolone, n (%)	20 (64.52%)	29 (71.87%)	NA
Phosphor and magnesium, n (%)	1 (3.23%)	2 (6.84%)	NA
Calcitriol, n (%)	1 (3.23%)	2 (6.84%)	NA
Insulin aspart, n (%)	2 (6.45%)	3(9.98%)	NA
Beta-blocker, n (%)	3 (9.68%)	3(9.98%)	NA
Vitamin B complex, n (%)	2 (6.45%)	4 (12.87%)	NA
Desal, n (%)	1 (3.23%)	2(6.84%)	NA
Entinostat, n (%)	1 (3.23%)	1 (3.41%)	NA
EPO, n (%)	1 (3.23%)	1 (3.41%)	NA
Prednol, n (%)	1 (3.23%)	1 (3.41%)	NA
Dilatrend, n (%)	1 (3.23%)	1 (3.41%)	NA
Diltiazem, n (%)	2 (6.45%)	1 (3.41%)	NA

**Table 2:** Laboratory investigations of the patients hospitalized for COVID-19 COVID-19, coronavirus disease 2019; SD, standard deviation; ESR, erythrocyte sedimentation rate; CRP, C-reactive protein; AST, aspartate aminotransferase; ALT, alanine aminotransferase; LDH, lactate dehydrogenase; WBC, white blood cell

Clinical characteristics	Hospitalized for COVID-19, n	Mean	SD	Median	Minimum	Maximum
Drug level, tacrolimus	31	5.45	1.99	5.4	3.07	12.05
Neutrophil (%)	31	87.82	62.81	80.4	49.4	417
Lymphocyte (%)	31	15.2	10.7	13	0.5	36.8
Hemoglobin (g/L)	31	7.34	4.68	7.7	0.72	18.4
Creatine kinase (U/L)	31	17.1	25.3	12.3	8.3	153
ESR (mm/h)	31	102.32	146.4	55	8	634
CRP (mg/L)	31	42.59	65.58	22.77	0.43	339.14
Albumin (g/L)	30	28.45	13.89	34	40.1	46
AST (U/L)	30	30	29.2	21	5	150
ALT (U/L)	30	41.4	54.5	22.5	8	246
LDH (U/L)	31	317	252	259	56	1339
Total bilirubin (mg/dL)	30	0.61	0.39	0.55	0.2	1.88
Ferritin (ng/mL)	27	377.72	417.67	236.4	15.1	1650
WBC	31	8.47	4.79	7.45	2.09	22.5

**Table 3:** Laboratory investigations of the patients with COVID-19 treated as outpatients COVID-19, coronavirus disease 2019; SD, standard deviation; ESR, erythrocyte sedimentation rate; CRP, C-reactive protein; AST, aspartate aminotransferase; ALT, alanine aminotransferase; LDH, lactate dehydrogenase; WBC, white blood cell; NA, not applicable

Clinical characteristics	Outpatient COVID-19 treatment, n	Mean	SD	Median	Minimum	Maximum
Drug level, tacrolimus	42	9.23	0.05	8.32	6.00	16.95
Neutrophil (%)	42	67.51	11.63	68.60	49.30	99.10
Lymphocyte (%)	42	21.8	9.5	21.5	0.0	44.9
Hemoglobin (g/L)	42	7.67	3.34	7.50	0.40	16.20
Creatine kinase (U/L)	42	12.0	2.8	12.4	7.0	16.8
ESR (mm/h)	42	3.72	3.33	2.86	0.67	15.20
CRP (mg/L)	0	NA	NA	NA	NA	NA
Albumin (g/L)	42	4.11	0.73	4.14	1.82	5.29
AST (U/L)	41	16.4	9.9	14.5	7.8	63.0
ALT (U/L)	41	21.4	15.5	19.0	3.0	89.0
LDH (U/L)	35	222	75	217	17	477
Total bilirubin (mg/dL)	42	0.54	0.71	0.34	0.02	3.96
Ferritin (ng/mL)	20	229.95	197.69	160.00	8.90	730.00
WBC	41	8.50	4.81	7.68	1.68	33.42

**Table 4:** Serum tacrolimus levels of hospitalized and outpatient-treated renal transplantation patients with COVID-19 COVID-19, coronavirus disease 2019; SD, standard deviation; OR, odds ratio; CI, confidence interval

Hospitalization status	Serum tacrolimus level, n	SD	Median	Min	Max	p-value	OR	95% CI for OR	
								Lower	Upper
Hospitalized	31	1.99	5.40	3.07	12.05	0.002	1.400	1.102	1.779
Outpatient	42	0.05	8.32	6.00	16.95	0.002			

There was no statistically significant difference in mean age between COVID-19-positive patients and the control group (p > 0.05). However, gender distribution differed significantly; there were more male than female participants among the COVID-19-positive patients, with a higher proportion of males hospitalized (p < 0.05; Table 1).

No statistically significant difference was observed between the hospitalized and outpatient-treated COVID-19-positive transplantation recipients patients in terms of the routine biochemistry results and the drugs used (p > 0.05; Tables 3, 4). However, serum tacrolimus levels were significantly higher in outpatient-treated patients, 1.4 times higher than in hospitalized patients (p < 0.05; Figure 1).

No significant correlation was found between donor-recipient HLA match and CO-RADS evaluations for hospitalized patients. Lung involvement with a CO-RADS score of 5 was observed in patients with 1A-2A, 2DR, and 2A, 1B, 1DR matches. Among patients with a 1DR match, 35% had a CO-RADS score of 4, and 75% had a score of 5. In those with a 1A, 1B, 1DR match, 35% had CO-RADS 2, and 55% had scores ranging from 3 to 5. Furthermore, 55% of patients with a 1A, 1DR match showed lung involvement with a CO-RADS 3, and 45% had a CO-RADS 4. Among those with a 1B, 1DR match, 40% had a CO-RADS 3, and 60% had a CO-RADS 5. Patients with a 1B, 2DR match showed 85% at CO-RADS 2 and 25% at CO-RADS 5. For those with a 2A, 2B, 2DR match, 55% showed lung involvement with a CO-RADS 3 and 45% with a CO-RADS 4 (Tables 5-11).

**Table 5:** Comparison of HLA allele frequencies between the COVID-19 patient groups and the control group HLA, human leukocyte antigen; COVID-19, coronavirus disease 2019

HLA allele	Hospitalized for COVID-19		Control (n = 50)	p-value
	Yes (n = 31)	No (n = 42)		
A*01	3 (12.90%)	8 (3.36%)	7 (14%)	0,551
A*02	11 (45.16%)	17 (7.56%)	16 (32%)	0.446
A*03	8 (25.81%)	10 (4.42%)	13 (26%)	0.967
A*11	1 (3.23%)	9 (3.78%)	19 (36%)	0.009
A*23	3 (9.68%)	3 (1.26%)	3 (6%)	0.829
A*24	10 (35.48%)	16 (7.98%)	24 (48%)	0.681
A*26	2 (6.45%)	4 (1.68%)	4 (8%)	1.000
A*29	0	2 (0.84%)	1 (2%)	0.615
A*30	5 (16.13%)	3 (1.26%)	3 (6%)	0.315
A*31	2 (3.23%)	1 (0.42%)	0	0.117
A*32	1 (3.23%)	2 (0.84%)	3 (6%)	1.000
A*33	1 (3.23%)	0	2 (4%)	0.473
A*66	1 (3.23%)	0	0	0.252
A*68	4 (12.90%)	4 (1.68%)	5 (10%)	0.869
A*69	0	1 (0.42%)	0	0.593

**Table 6:** Comparison of HLA allele frequencies between the COVID-19 kidney transplant recipient patients Adjusted logistic regression model for COVID-19 infection HLA, human leukocyte antigen; COVID-19, coronavirus disease 2019; OR, odds ratio; NA, not applicable

HLA allele	Hospitalized for COVID-19				OR	p-value
	Yes (n = 31)		No (n = 42)			
	N	%	N	%		
A*01	3	9.7%	8	19.0%	2.196 (0.532-9.068)	0.438
A*02	11	35.5%	17	40.5%	1.236 (0.474-3.228)	0.665
A*03	8	25.8%	10	23.8%	0.898 (0.307-2.627)	0.845
A*11	1	3.2%	9	21.4%	8.182 (0.978-68.459)	0.059
A*23	3	9.7%	3	7.1%	0.718 (0.135-3823)	0.694
A*24	10	32.3%	16	38.1%	1.292 (0.486-3.434)	0.607
A*26	2	6.5%	4	9.5%	1.526 (0.261-8.915)	1.000
A*29	0	0.0%	2	4.8%	NA	0.505
A*30	5	16.1%	3	7.1%	0.400 (0.088-1.820)	0.403
A*31	2	6.5%	1	2.4%	0.354 (0.031-4.086)	0.571
A*32	1	3.2%	2	4.8%	1.500 (0.130-17.325)	1.000
A*33	1	3.2%	0	0.0%	NA	0.425
A*66	1	3.2%	0	0.0%	NA	0.425
A*68	4	12.9%	4	9.5%	0.711 (0.163-3.094)	0.938
A*69	0	0.0%	1	2.4%	NA	1.000

**Table 7:** Comparison of HLA allele frequencies between the COVID-19 patient groups and the control group HLA, human leukocyte antigen; COVID-19, coronavirus disease 2019

HLA allele	Hospitalized for COVID-19		Control (n = 50), n (%)	p-value
	Yes (n = 31), n (%)	No (n = 42), n (%)		
B*07	0	0	2 (4%)	0.340
B*08	4 (12.90%)	1 (2.38%)	0	0.011
B*13	2 (6.45%)	1 (2.38%)	5 (8%)	0.546
B*14	1 (3.23%)	0	0	0.252
B*15	1 (3.23%)	2 (4.76%)	3 (6%)	1.000
B*18	1 (3.23%)	4 (9.52%)	4 (8%)	0.620
B*27	2 (6.45%)	2 (4.76%)	4 (8%)	0.898
B*34	0	1 (2.38%)	0	0.593
B*35	10 (41.94%)	20 (54.76%)	21 (42%)	0.392
B*38	2 (9.68%)	4 (9.52%)	4 (8%)	1.000
B*39	0	2 (4.76%)	2 (4%)	0.677
B*40	0	3 (7.14%)	3 (6%)	0.367
B*41	1 (3.23%)	3 (7.14%)	2 (4%)	0.761
B*44	4 (12.90%)	6 (14.29%)	5 (10%)	0.781
B*49	3 (12.90%)	5 (11.90%)	7 (14%)	0.939
B*50	2 (6.45%)	2 (4.76%)	3 (6%)	1.000
B*51	9 (32.26%)	14 (33.33%)	18 (36%)	0.925
B*52	4 (12.90%)	3 (7.14%)	12 (24%)	0.086
B*53	1 (3.23%)	1 (2.38%)	1 (2%)	1.000
B*54	0	2 (4.76%)	0	0.177
B*55	3 (9.68%)	4 (9.52%)	2 (4%)	0.502
B*57	1 (3.23%)	0	2 (4%)	0.473
B*58	0	1 (2.38%)	1 (2%)	1.000
B*60	1 (3.23%)	0	0	0.252
B*73	0	0	1 (2%)	1.000

**Table 8:** Comparison of HLA allele frequencies between the COVID-19 kidney transplant recipient patients Adjusted logistic regression model for COVID-19 infection HLA, human leukocyte antigen; COVID-19, coronavirus disease 2019; OR, odds ratio; NA, not applicable

HLA allele	Hospitalized for COVID-19				OR	p-value
	Yes (n = 31)		No (n = 42)			
	N	%	N	%		
B*07	0	0%	0	0%	NA	NA
B*08	4	12.9%	1	2.4%	0.165 (0.017-1.553)	0.156
B*13	2	6.5%	1	2.4%	0.354 (0.031-4.086)	0.571
B*14	1	3.2%	0	0.0%	NA	0.425
B*15	1	3.2%	2	4.8%	1.500 (0.130-17.325)	1.000
B*18	1	3.2%	4	9.5%	3.158 (0.335-29.752)	0.387
B*27	2	6.5%	2	4.8%	0.725 (0.096-5.451)	1.000
B*34	0	0.0%	1	2.4%	NA	1.000
B*35	10	32.3%	20	47.6%	1.909 (0.726-5.018)	0.187
B*38	2	6.5%	4	9.5%	1.526 (0.261-8.915)	1.000
B*39	0	0.0%	2	4.8%	NA	0.505
B*40	0	0.0%	3	7.1%	NA	0.257
B*41	1	3.2%	3	7.1%	2.308 (0.228-23.311)	0.632
B*44	4	12.9%	6	14.3%	1.125 (0.289-4.383)	1.000
B*49	3	9.7%	5	11.9%	1.261 (0.278-5.728)	1.000
B*50	2	6.5%	2	4.8%	0.725 (0.096-5.451)	1.000
B*51	9	29.0%	14	33.3%	1.222 (0.447-3.344)	0.696
B*52	4	12.9%	3	7.1%	0.519 (0.107-2.509)	0.448
B*53	1	3.2%	1	2.4%	0.732 (0.044-12.172)	1.000
B*54	0	0.0%	2	4.8%	NA	0.505
B*55	3	9.7%	4	9.5%	0.982 (0.203-4.744)	1.000
B*57	1	3.2%	0	0.0%	NA	0.425
B*58	0	0.0%	1	2.4%	NA	1.000
B*60	1	3.2%	0	0.0%	NA	0.425

**Table 9:** Comparison of HLA allele frequencies between the COVID-19 patient groups and the control group HLA, human leukocyte antigen; COVID-19, coronavirus disease 2019

HLA allele	Hospitalized for COVID-19		Control (n = 50), n (%)	p-value
	Yes (n = 31), n (%)	No (n = 42), n (%)		
DR*01	5 (16.13%)	9 (21.43%)	9 (18%)	0.837
DR*03	6 (22.58%)	3 (7.14%)	6 (12%)	0.316
DR*04	14 (48.38%)	16 (47.62%)	16 (32%)	0.376
DR*07	4 (12.90%)	8 (19.05%)	5 (10%)	0.427
DR*08	0	0	7 (12%)	0.009
DR*09	2 (6.45%)	1 (2.38%)	2 (4%)	0.731
DR*10	1 (3.23%)	1 (2.38%)	1 (2%)	1.000
DR*11	10 (35.48%)	8 (19.05%)	18 (36%)	0.246
DR*12	1 (3.23%)	0	3 (6%)	0.363
DR*13	4 (12.90%)	11 (26.19%)	9 (36%)	0.245
DR*14	2 (6.45%)	3 (7.14%)	7 (14%)	0.723
DR*15	6 (19.35%)	13 (38.10%)	15 (30%)	0.488
DR*16	0	3 (7.14%)	2 (4%)	0.367

**Table 10:** Comparison of HLA allele frequencies between the COVID-19 kidney transplant recipient patients Adjusted logistic regression model for COVID-19 infection HLA, human leukocyte antigen; COVID-19, coronavirus disease 2019; OR, odds ratio; NA, not applicable

HLA allele	Hospitalized for COVID-19				OR	p-value
	Yes (n = 31)		No (n = 42)			
	N	%	N	%		
DR*01	5	16.1%	9	21.4%	1.418 (0.424-4.746)	0.570
DR*03	6	19.4%	3	7.1%	0.321 (0.073-1.400)	0.227
DR*04	14	45.2%	16	38.1%	0.747 (0.291-1.918)	0.544
DR*07	4	12.9%	8	19.0%	1.588 (0.432-5.841)	0.484
DR*08	0	0%	0	0%	NA	NA
DR*09	2	6.5%	1	2.4%	0.354 (0.031-4.086)	0.571
DR*10	1	3.2%	1	2.4%	0.732 (0.044-12.172)	1.000
DR*11	10	32.3%	8	19.0%	0.494 (0.168-1.451)	0.196
DR*12	1	3.2%	0	0.0%	NA	0.425
DR*13	4	12.9%	11	26.2%	2.395 (0.683-8.404)	0.165
DR*14	2	6.5%	3	7.1%	1.115 (0.175-7.112)	1.000
DR*15	6	19.4%	13	31.0%	1.868 (0.618-5.641)	0.264
DR*16	0	0.0%	3	7.1%	NA	0.257

**Table 11:** Comparison of HLA match rates between the COVID-19 kidney transplant recipients HLA, human leukocyte antigen; COVID-19, coronavirus disease 2019; NA, not applicable

HLA allele	Recipient-donor match	Hospitalized for COVID-19	
		Yes (n = 31), n (%)	No (n = 42), n (%)
HLA-A	NA (mismatch)	8 (25.81%)	17 (40.48%)
	One allele match	18 (52.6%)	22 (58.38%)
	Two allele match	4 (12.90%)	4 (9.52%)
HLA-B	NA (mismatch)	10 (32.26%)	19 (45.24%)
	One allele match	17 (54.84%)	21 (50%)
	Two allele match	4 (12.90%)	3 (7.14%)
HLA-DR	NA (mismatch)	2 (6.45%)	7 (16.67%)
	One allele match	22 (70.975)	23 (54.76%)
	Two allele match	7 (22.58%)	13 (30.95%)
Total match	1/6 match	4 (12.90%)	6 (14.29%)
	2/6 match	7 (22.58%)	11 (26.19%)
	3/6 match	11 (35.48%)	17 (40.48%)
	4/6 match	4 (12.90%)	4 (9.52%)
	5/6 match	0 (0%)	1 (2.38%)
	6/6 match	2 (6.45%)	1 (2.38%)

The distribution and percentages of HLA allele frequencies were analyzed for each recipient and compared between groups. The presence of the HLA-B*08 allele was associated with an increased likelihood of hospitalization (p = 0.011), suggesting it may contribute to disease susceptibility. Conversely, the HLA-A*11 allele frequency was significantly higher in the control group and outpatient-treated patients (p = 0.009; odds ratio = 8; 95% confidence interval = 0.978-68.459; p = 0.059) (Table 5-10).

When examining the HLA match ratios, higher percentages of HLA-A non-match/1A match, HLA-B non-match/1B match, and HLA-DR non-match/2DR match were observed in the outpatient group. The prevalence of a 3/6 match was higher among outpatients, while no significant differences were found in 1/6, 2/6, 4/6, 5/6, and 6/6 matches between the groups (p > 0.05). No significant differences were noted in HLA match ratios related to hospitalization status (p > 0.05) (Table 11).

## Discussion

This study investigates factors contributing to the spread and varying clinical symptoms of COVID-19, focusing on the role of gender and age in disease progression. We analyzed age, gender, etiology, recipient HLA typing, donor-recipient compatibility, the impact of immunosuppressant drug levels on COVID-19 infection, and the potential relationship between transplant HLA compatibility and lung involvement scoring in hospitalized kidney transplant recipients.

Consistent with Turkish epidemiological data, our findings indicate that males were more adversely affected by COVID-19 than females. No significant age differences were observed between hospitalized and outpatient COVID-19-positive patients [12,13]. Zhong et al. reported on two transplant recipients with COVID-19 who had differing treatments and prognoses, suggesting implications for managing immunosuppression in such patients [14]. Yin et al. noted that patients with better prognoses often continued tacrolimus, whereas those requiring more intensive care ceased using it post-diagnosis, highlighting a potential selection bias in studies relating tacrolimus use to prognosis [15].

A study involving 243 adult liver transplant recipients with symptomatic COVID-19 across 36 centers in nine countries found that the risk of mortality was elevated in patients over age 70 and those with renal dysfunction or diabetes [16]. Our study also found a significant correlation between serum tacrolimus levels and patient management; outpatient cases had 1.4-fold higher levels than hospitalized patients who received reduced doses.

The role of HLA class I and II genes in the immune response to COVID-19 was explored through genotyping research, revealing a potential protective or susceptibility role of specific HLA alleles [17]. In-silico analyses and other studies have identified the HLA-A11 allele as a significant factor in COVID-19 outcomes. For instance, a Spanish study linked the HLA-A11 allele with increased hospitalization and mortality rates among COVID-19 patients, a trend also observed in other infections like hepatitis B and active pulmonary tuberculosis [18]. High-resolution sequencing in Tokyo identified a significant correlation between HLA-A*11:01:01:01 and COVID-19 severity [19], further supported by its known sensitivity to influenza A H1N1.

Our results are consistent with previous findings that show significant differences in the HLA-A11 allele between individuals in the control group and outpatient transplant recipients [20]. The distinct difference in HLA-A11 among outpatient patients suggests that this allele may have a potentially protective effect. We hope that these positive findings from our study when supported by research conducted on larger and more diverse populations could lead to more definitive conclusions about the effects of HLA-A11 on COVID-19 [20].

Early studies during the COVID-19 pandemic utilized bioinformatics to explore HLA’s role in the disease, estimating HLA binding affinity to SARS-CoV-2 peptides. These estimates indicated a high affinity for several HLA alleles, pointing to the need for comprehensive global studies to elucidate the relationship between HLA variations and COVID-19 outcomes [21-23]. A recently expanded genome-wide association study meta-analysis did not find a significant impact of the HLA locus on COVID-19 severity but did identify specific alleles as protective or risk factors [24].

Our study has several important limitations. The small sample size makes it difficult to generalize the results and fully understand the impact of COVID-19 on clinical outcomes and disease severity. Additionally, the single-center and retrospective design of our study limits the applicability of our findings to broader and more diverse populations. The homogeneity of our study population may not reflect the variability observed in larger, more diverse groups. Furthermore, HLA typing was limited to the available samples, and not all possible HLA alleles were analyzed, which may overlook additional genetic factors that could influence disease susceptibility and severity. Despite these limitations, our study makes a valuable contribution to the existing literature and lays an important foundation for future research.

## Conclusions

This study investigated the susceptibility of HLA alleles to COVID-19 infection and their potential protective effects. We observed statistically significant associations between specific HLA alleles and COVID-19 infection rates. Additionally, the impact of COVID-19 was assessed by analyzing patient demographics and drug levels used in treatment. Geographic variations in HLA allele distributions may explain the diverse findings observed globally, highlighting the need for more comprehensive international studies involving organ transplant recipients from diverse populations. Our findings suggest that certain HLA alleles may influence susceptibility to COVID-19 or provide some level of protection. Notably, the significant difference in the HLA-A*11 allele between the control group and outpatient-treated transplant recipients indicates that this allele may offer protective benefits against COVID-19. This emphasizes the importance of considering genetic factors in managing and understanding COVID-19 among transplant recipients.
